# *Moringa oleifera* fruit extract as a potential antioxidant against liver injury by 2-Nitropropane induction in obese male mice model: pre-clinical study

**DOI:** 10.12688/f1000research.121695.2

**Published:** 2024-02-12

**Authors:** Maria Selvester Thadeus, Tiwuk Susantiningsih, Hikmah Muktamiroh, Cut Fauziah, Mila Citrawati, Agneta Irmarahayu, Sri Wahyuningsih, Yanti Harjono Hadiwiardjo, Hany Yusmaini, Meiskha Bahar, Fajriati Zulfa, Diana Agustini, Aulia Chairani

**Affiliations:** 1 Medical Faculty, UPN Veteran Jakarta, Jakarta, Indonesia, 12450, Indonesia; 2 Patologi Anatomi, UPN Veteran Jakarta, Jakarta, DKI Jakarta, 12450, Indonesia

**Keywords:** 2-Nitropropane induction, liver injury, Moringa oleifera fruit extract, obese male mice model, potential antioxidant

## Abstract

**Background:** *Moringa oleifera* fruit extract contains beneficial chemical compounds. This study was conducted to observed the power of antioxidant against liver injury by 2-Nitropropane induction in an obese male mice model.

**Methods:** This research was *in vivo* laboratory experimental study with a post-test control design group only. The population was obese male mice models, Swiss strain, aged 6–8 weeks, weighing between 60–80 gr. The research sample was determined by Federer's formula for a complete randomized design experimental test, group N (control), O1 (induced by 2-Nitropropane intraperitoneal (i.p) once), O2 (induced by 2-Nitropropane i.p twice), P1 (induced by 2-Nitropropane i.p. once and gavage with *M. oleifera* fruit extract 500mg/kg bodyweight (BW) once a day), P2 (induced by 2-Nitropropane i.p. twice and gavage of *M. oleifera* fruit extract 500mg/kg BW once a day), and P3 (induced by 2-Nitropropane i.p. twice and gavage of vitamin C 500mg/kg BW once a day). Antioxidant potential parameters were measured by levels of malondialdehide (MDA), glutation (GSH), 8-hydroxy-2'-deoxyguanosine (8-OHdG), catalase activity, manganese superoxide dismutase (MnSOD), serum glutamic pyruvic transaminase (SGPT), serum glutamic oxaloacetic transaminase (SGOT). This research was held at the Biochemistry laboratory of Medicine Faculty, UPN Veteran Jakarta in May–September 2020. Analysis was carried out using SPSS version 20.0. The parameters were tested using ANOVA.

**Results:** MDA levels decreased, GSH increased, 8-OHdG decreased, catalase activity increased, MnSOD activity increased and SGOT, SGPT levels decreased. *M. oleifera* fruit extract was statistically proven to be a candidate for potential antioxidant against liver injury of 2-Nitropropane induction in obese male mice model.

**Conclusions:** *M. oleifera* fruit extract was statistically evident as an antioxidant substance that reduces oxidative stress in acute liver injury caused by 2-Nitropropane induction.

## Introduction

Indonesia has abundant natural wealth, spread from the west to the east. ^1^ One of the natural resources is plants as a source of traditional medicine. *Moringa oleifera* fruit is the Moringa plant's fruit, and contains beneficial chemical compounds. ^2^ The vitamin content in *M. oleifera* includes vitamin C, vitamin A, vitamin B1 and B2. The antioxidant content includes flavonoids, quercetin, isothiocyanates and glucosinolates. ^3^^,^^4^ In addition, Moringa fruit also has anti-sensitivity, anti-inflammatory, and anti-rheumatic, anti-hypertensive, anti-diabetic, anti-cancer properties. ^3^^–^^5^

*M. oleifera* has antimicrobial, antibacterial, antifungal and anthelmintic properties. ^5^^–^^9^

Antioxidants are chemical ingredients that can donate one or more electrons to free radicals so that free radicals can be mutated. ^7^^,^^8^ There are two antioxidants: natural antioxidants and synthetic antioxidants. Synthetic antioxidants are industrial products. ^10^^–^^12^

Natural antioxidants can protect the body against damage caused by reactive oxygen species that can inhibit the occurrence of degenerative diseases and can inhibit lipid peroxidase in the body. ^13^ Vitamin C as an antioxidant exogen can be used to reduce oxidative stress. It can be an effective antioxidant in inhibiting free radicals with a suitable dose and its chemically capable of reacting with most radicals free and oxidants in the body. ^14^^,^^15^ In 2016, obesity was estimated to exceed 250 million people worldwide. ^16^ Obesity was the third leading cause of cancer after tobacco and alcohol. ^15^ Experts estimate that obesity causes about 20% of all cancer cases. ^14^^,^^15^^,^^17^

Epidemiological studies show that obesity was associated with an increased risk of cancer. ^16^^,^^18^ One of the cancers thought to occur due to obesity was hepatocellular carcinoma. ^13^^,^^17^^,^^19^ Obesity causes dysregulation of growth signals such as increased insulin and leptin. ^20^^–^^22^ Obesity causes angiogenesis, tumorigenesis and chronic inflammation. ^15^^,^^23^ Experts suspect obesity is also associated with the incidence of hepatocellular cancer. ^15^^,^^24^^,^^25^ The chemical 2-Nitropropane can induce hepatocellular. ^26^ 2-Nitropropane (2NP) can trigger the mechanism of hepatocarcinogenesis. ^17^^,^^24^^,^^27^

Research by Metwally *et al.* (2017) in obese female rats given *M. oleifera* at a dose of 600 mg/kgBW orally for 12 weeks is a good candidate for therapy for symptoms of metabolic syndrome. ^8^

There have been many studies reporting on *M. oleifera* fruit. However, only a few studies have reported on the potential of *M. oleifera* fruit extract as a potent antioxidant against liver injury in the early events of chemically induced carcinogenesis, such as 2-Nitropropane in obese male mice models. Can *M. oleifera* fruit extract treat liver injury by 2-Nitropropane induction in an obese male mice model? This research observed the compound antioxidant of *M. oleifera* fruits extract against liver injury by 2-Nitropropane induction in an obese male mice model. *M oleifera* fruits extract can treat the liver injury by 2-Nitropropane induction in an obese male mice model. ^3^^,^^5^

## Methods

### Ethical considerations

This study protocol was reviewed and approved by The Health Research Ethics Committee (HREC) Pembangunan Nasional Veteran University Jakarta, approval number B/1822/4/2019/KEPK on April 23 ^th^ 2019. This study was held May–September 2020, and all authors provide a statement indicating that all efforts were made to ameliorate harm to animals. Researchers agreed to use the minimum number of experimental animals using the Ferderer formula. The experimental animals were treated according to the 5 F principle. The experimental animals were terminated with ketamine xylazine anesthesia, then buried.

### Research design

The research design was an experimental laboratory study with a post-test control group only design. The indicators seen on antioxidant potential were by seeing malondialdehyde (MDA), glutathione (GSH), 8-Hydroxy-2′-deoxyguanosine (8-OhdG), catalase activity, manganese superoxide dismutase (MnSOD), serum glutamic pyruvic transaminase (SGPT), and serum glutamic oxaloacetic transaminase (SGOT). The research location was in the Biochemistry Laboratory Faculty of Medicine Universitas Pembangunan Nasional Veteran Jakarta. The population was male obese mice models Swiss strain aged 6–8 weeks weighing between 60–80gr obtained from the Animal Laboratory of University Institut Pertanian Bogor, Bogor West Java. Determination of the research sample using the Federer formula for a completely randomized design experimental test.

Inclusion criteria: mice in healthy condition (active and no anatomical abnormalities), weight 60–80 gr, age 6–8 weeks, line: Swiss, gender: male. Exclusion criteria: sick and die during the process of adaptation and treatment.

Materials: Cages, food and drink container, scales, analytical scales, logbook and stationery, S-30 spectrophotometer ThermoFisher ^®^, homogenizer Tokyo Rikaikai ^®^, micropipette, minor surgical instruments, one cc syringe, three cc syringe, vacutainer, micro tip, microtubes vortex, microtube rack, and cuvettes. Materials: high fat high protein feed, 2-Nitropropane (2NP) liquid From Aldrich ^®^, MDA Assay kit Elabscience ^®^, GSH Assay kit Elabscience ^®^, 8-OHdG Assay kit Elabscience ^®^, Catalase activity Assay kit Elabscience ^®^, MnSOD kit RanSOD ^®^, SGPT Assay kit Elabscience ^®^, SGOT Assay kit Elabscience ^®^, ice, Ketamine-xylazine liquid AgrovetMarket ^®^.

### Research procedure

Mice were obtained from the Animal Laboratory of University IPB Bogor, the health of the mice was monitored every day, and their body weight was weighed before conducting the study. After going through a laboratory adaptation process for seven days, obesity was induced in mice aged 4–5 weeks by feeding them high-fat diets for 4 weeks until the average body weight of obese mice was 60–80g. Then the mice were placed in a plastic cage with a lid made of ram wire and covered with husk, fed with high-fat feed pellets and drinking water was given *ad libitum.* The cage environment was made so that it was not humid, with adequate ventilation and adequate lighting where the light time is 14 hours and the dark was 10 hours.

The sample consisted of 36 Swiss obese male mice models divided into six groups. Each group consisted of six mice: group N (obese mice control), O1 (obese mice induced of 2-Nitropropane 20 mg/kgBW intraperitoneal (i.p.) once), O2 (obese mice induced of 2-Nitropropane 20mg/kgBW i.p. twice), P1 (obese mice induced of 2-Nitropropane 20 mg/kgBW i.p. once and gavage with *Moringa oleifera* fruit extract 500 mg/kgBW once a day), P2 (obese mice induced of 2-Nitropropane 20 mg/kgBW i.p. twice and gavage of *Moringa oleifera* fruit extract 500 mg/kgBW once a day), and P3 (obese mice induced of 2-Nitropropane 20 mg/kgBW i.p. twice and gavage of vitamin C 500 mg/kgBW once a day). After induction, the mice were treated with betadine.

This study using obesity models of mice; a possible thing that can happen is the sudden death of mice which causes the need to use new mice so the number of mice increases. Each mouse that has undergone surgery and liver removal will be buried. The mice were well cared for, and fed according to the standard. After completing the research, the mice were necropsied and buried. During the study there were no sick mice or mice that had adverse effects, so no mice were excluded.

After 10 weeks, the mice were fasted, then necroption under anesthesia: xylazine 0.1 mg/kgBW + ketamine 0.03 mg/kgBW by i.p., then euthanized. Prior to necropsy, the mice were anesthetized using ketamine xylazine until completely asleep and then dissected. The blood samples were taken from the heart’s left ventricle with a 3cc syringe by cardiac puncture. The blood was placed in a vacutainer, and the serum was stored at -80°C. The liver was taken and weighed then examined for biochemical markers. The dissected mice will be put into a special animal bag, then buried properly. The liver tissue will be measured for MDA, GSH, 8-OHdG, Catalase activity, MnSOD, SGPT, and SGOT levels.

### The preparation of induction 2-Nitropropane

The dose of 2-Nitropropane (130263-5ml Aldrich ^®^) of 0.02 mg/kgBW was given intraperitoneally to induce oxidative stress and liver injury in male obese mice models. ^27^

### *M. oleifera* fruits extraction

The research used *M. oleifera* fruit extract from Lampung Province was a concentrated preparation obtained by removing the active ingredients of *M. oleifera* fruits. A total of 500 g of dried simplicia *M. oleifera* fruits with 70% ethanol solvent maceration got 55.2 g of *M. oleifera* fruits to extract.

### Liver homogenate using a homogenizer

A homogenate was made by putting 100 mg of mice liver tissue into a 1.5 ml Eppendorf tube, then adding 1 ml of phosphate buffered saline (PBS) (0.01 M pH 7.4). This was crushed using a homogenizer for 90 seconds (maximum time) at a speed of 3500 rpm (the crushed samples were placed in the ice floes until they are all crushed) using Tokyo Rikaikai homogenizer. Next, the Eppendorf tube was inserted and centrifuged for 10 minutes at 3500 rpm. To obtain the supernatant from liver tissue, the supernatant was taken and transferred to a new Eppendorf tube. After that, the Eppendorf tube was put in the refrigerator with a minimum temperature of -20°C overnight.

### MDA Level examined

Examination of liver MDA levels using the Will method ^28^ from Assay kit Elabscience ^®^ at the Biochemistry Laboratory, Faculty of Medicine, University of Indonesia. A total 10 μL MDA Color Reagent solution was added into each well of MDA Standard, Blank Control, and Test Samples. The reaction mixture was incubated at room temperature for 10–30 minutes and 40 μL of Reaction Solution was added to make the total assay volume to 100 μL/well. The final reaction mixture was incubated at room temperature for 30–60 minutes. The end-point absorbance was measured with an absorbance plate reader with path-check correction at optical density (OD) 695–700 nm. Absorption readings were taken using a spectrophotometer (Thermo scientific ^®^ ab233471 Lipid Peroxidation (MDA) Assay Kit (Colorimetric)). Furthermore, to get the concentration results is to plot the absorbance data of the sample into a standard curve. ^23^

### GSH levels were examined

Homogenate supernatant as much as 40 μL entered a 1.5 ml tube, then 210 μL of 5% trichloroacetic acid was added and mixed using a 3–5 second vortex. Then the solution was centrifuged at 5000 rpm for 10 minutes, then take the supernatant and transferred to a 2 ml Eppendorf tube. Then 800 μL of PBS pH 8.0 was added and mixed by vortex until homogenous. After that, 25 μL 5,5′-Dithiobis 2-nitrobenzoic acid (DTNB) reagent was added so that the yellow-colored compound benzoate would be formed due to the binding of the hydryl sulfur (SH) group by DTNB. This was then incubated at room temperature for 1 hour in a dark room, then the absorbance was read using a spectrophotometer (Thermo scientific ^®^) at the wavelength (λ) = 412 nm. All samples were measured by using the Duplo principle (measurement twice); to avoid errors in calculations. ^23^^,^^28^

### 8-OHdG level examined

We took 10 mg of homogenate dissolved in 0.1M phosphate buffer pH 7.5 was homogenized and then centrifuged at 1000 × g for 10 minutes. The supernatant was purified using a Deoxyribonucleic acid (DNA) (Quick-DNATM MiniPrep Plus) extraction kit. The DNA was digested using nuclease P1 following the manufacturer's instructions. The pH was adjusted to 7.5–8.5 using 1 M Tris buffer. One unit per 100 g of Alkaline phosphatase DNA was added and incubated at 37°C for 30 minutes. This was heated up for 10 minutes and placed on ice until used. ^29^^–^^31^

### MnSOD specific enzyme activity measurement

MnSOD activity was determined biochemically using the RanSOD ^®^ kit. It determined the total SOD activity utilizing the degree of inhibition of this formazan color as measured by a 505 nm spectrophotometer A. The sample was diluted 20 times using PBS 0.01 M pH 7. 25 μL of standard/diluted sample was put into the cuvette, then 20 μL of mixed substrate mixture was added, mixed well, and the xanthine oxidase enzyme was added. The researcher read the light absorption with a spectrophotometer at a wavelength of 505 nm in the first 30 seconds after the enzyme was added (A1) and 3 minutes later (A2). ^32^^,^^33^

### Catalase enzyme activity measurement

The liver of obese mice were weighed with an analytical balance of 100 mg and then inserted into a tube. Then PBS was added to as much as 1 mL/100 mg of the liver. ^32^^,^^34^

The liver and PBS tube were crushed using a homogenizer for 90 seconds. After thrashing, the tube was put into a centrifuge with 3500 rotations per minute for 10 minutes. The supernatant was then taken and moved it into a different tube. The tube was stored for one night at a temperature of -20 ^o^C. Catalase enzyme activity was expressed in U/mL. One unit means the amount of enzyme that catalyzes the reaction of 1 μM substrate per minute. Sample absorption value with formula:

$$\text{Complement activation}\;\left(\text{Unit}/\mathrm{mL}\right)=\frac{\left(\text{test absorption}-\text{blank absorption}\right)/\text{minute})}{\text{Molarity}\;H_{2}O_{2}\times \text{sample volume}}\times \text{dilution factor}$$

### SGPT and SGOT level measurement

The Eppendorf tube was taken from the refrigerator and 0.1 ml of the supernatant + 1 ml of the SGOT/SGPT reagent Tris was inserted into the cuvette and placed in the spectrophotometer (λ = 340 nm, temperature = 37°C) then mixed using a micropipette (the timer runs when the SGOT/SGPT reagent enters the first time). The start button on the spectrophotometer was clicked and the absorbance was read after 1 minute and 3 minutes. ^2^

### Analysis of data

Analysis was carried out using SPSS version 20. The tests were started with tests of normality and homogeneity. Parametric tests followed data that were normally distributed and homogeneous. Parametric test with one-way analysis of variance (ANOVA) and if different significantly, was followed by Bonferroni's post hoc test for multiple comparisons.

## Results

The results of the phytochemical from Balai Penelitian Tanaman Rempah dan Obat (BALITRO) Bogor showed the content of extract substances are alkaloids, tannins, flavonoids, and glycosides. The extracts have quercetin content, saponin and triterpenoid substances. The statistical analysis was conducted using SPSS version 20. Parametric test with ANOVA and Bonferoni’s post hoc test for multiple comparation.

### MDA and GSH levels

**Figure 1.**
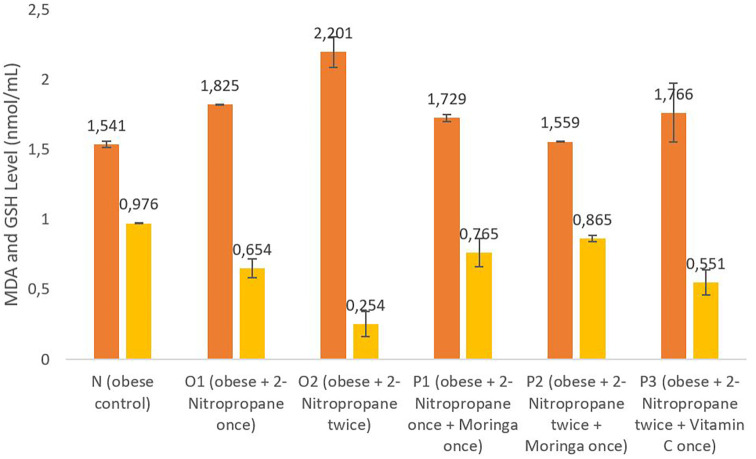
The malondialdehide (MDA) and glutathione (GSH) level. *M. oleifera* fruit extract as a candidate for potential antioxidant against for liver injury induced 2-Nitropropane in male obese mice models. The orange bars show MDA levels, the yellow bars show GSH levels.

Figure 1 shows that the *M. oleifera* fruits extract in groups P1 and P2 had an average MDA level of 1.729 nmol/mL and 1.559 nmol/mL, which was lower than group O1 and O2; the P3 group given vitamin C has an average MDA level of 1.766 nmol/mL, which was increased compared to the control group N and group O1, with an average MDA level of 1.541 nmol/mL and 1.825 nmol/mL. The GSH level of group O1 was decreased to 0.654 nmol/mL compared to the N group, O2 group. The group that was given *M. oleifera* fruits extract had an average GSH level of 0.765 nmol/mL, compared to group P1 and the group P2 of 0.865 nmol/mL. The GSH level of the P1 and P2 groups decreased compared to the O2 group. These data on the MDA level have been analyzed further using Post Hoc Test.

### 8-OHdG level, catalase activity and MnSOD activity

**Figure 2.**
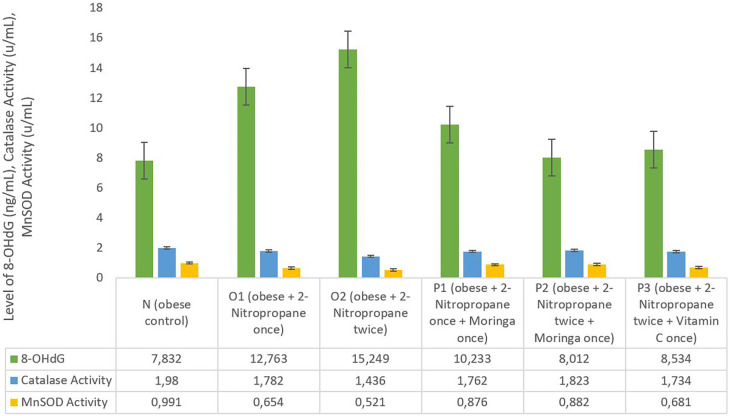
8-Hydroxy-2′-Deoxyguanosine (8-OHdG) and manganese superoxide dismutase (MnSOD) activity of *M. oleifera* fruit extract as a candidate for potential antioxidant against for liver injury induced 2-Nitropropane in male obese mice models.

The average liver 8-OHdG levels in groups O1 and O2 was increased (12.763 ng/mL and 15.249 ng/mL) compared to group N 7.832 ng/mL ( Figure 2). In group P1, P2 and P3 the average liver 8-OHdG was decreasing to 10.233 ng/mL, 8.012 ng/mL and 8.534 ng/mL. The catalase enzyme activity in the male obese mice model in group N shows 1.98 u/mL higher than all O1, O2, P1 P2 and P3 groups. In the O1 and O2 groups, there was a decrease in catalase activity of 1.782 u/mL and 1.436 u/mL compared to the other groups. The group given *M. oleifera* fruits extract had an average catalase activity enzyme level of 1.762 u/mL and 1.824 u/mL. The MnSOD activity level in group O1 was decreased compared to group N, and then more decreased in group O2, as seen on Figure 2, than other groups. The MnSOD activity enzyme level increased in the P1 (0.876 u/mL) and P2 (0.882 u/mL) groups which were given *M. Oleifera fruits* extract. The MnSOD activity enzyme level also increased in the P3 group (0.681 u/mL) which was given Vitamin C.

### SGOT and SGPT levels

**Figure 3.**
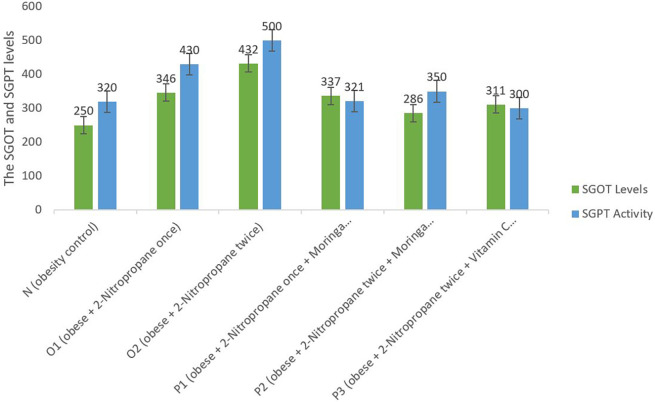
Serum glutamic oxaloacetic (SGOT) and serum glutamic pyruvic transaminase (SGPT) levels. *M. oleifera* fruit extract as a candidate for potential antioxidant against for liver injury induced 2-Nitropropane in male obese mice models.

SGOT and SGPT levels in the liver of each group showed a decrease in SGOT and SGPT levels in P1, P2 and P3 compared to O1 and O2 ( Figure 3). This figure showed a significant decrease in SGOT and SGPT levels in groups that were given *M. oleifera* fruits extract than groups N.

## Discussion

Moringa fruit obtained from the Moringa plant is a fruit with many benefits because it contains beneficial chemical compounds. The nutritional content in *M. oleifera* fruit includes vitamin C, vitamin A, vitamin B1 and vitamin B2, including flavonoids, quercetin, isothiocyanates and glucosinolates. ^6^^–^^8^ *M. oleifera* fruits have potential as antioxidants and prevent the spread of cancer cells, and can reduce cholesterol levels, carbohydrates, calcium, phosphorus, magnesium and potassium. ^1^^,^^9^^–^^11^ *M. oleifera* fruit extracts are effective as an antimicrobial, anti-sensitivity, anti-inflammatory, antirheumatic, anthelmintic, anti-cancer, antihypertensive, antibacterial, antifungal, antidiabetic, and antioxidant. ^2^^–^^4^

*M. oleifera* fruits extract contains flavonoids as antioxidants. ^2^^,^^35^ The antioxidant activity of flavonoids comes from their ability to donate hydrogen atoms and the ability to chelate metal. ^18^ *M. oleifera* fruits extract was also anti-obesity because it can down-regulate leptin and resistin mRNA expression while at the same time up-regulating the expression of adiponectin mRNA. ^8^ Vitamin C as an antioxidant agent given as a positive control in group P3 can be compared with *M. oleifera* fruits extract. The results of the research show that *M. oleifera* fruits extract have a significant difference as a candidate for antioxidant properties for decreasing oxidative level of induction of 2-Nitropropane in male obese mice models.

The results of this study showed that there was a decrease in MDA levels in P1 and P2 groups that were given *M. oleifera* fruits extract. The level of GSH increased in the groups O1 and O2. So, it can be concluded that *M. oleifera* fruits extract can inhibit oxidative stress due to the induction of 2-Nitropropane. As a positive control in group P3, vitamin C also does the same as an antioxidant exogen. So, this study proved that *M. oleifera* fruits extract has the ability as an exogenous antioxidant.

The level of 8-OHdG also decreased when groups P1 and P2 were given *M. oleifera* fruits extract compared to groups O1 and O2. So, we can see that the administration of *M. oleifera* fruits extract can repair damaged cells caused by 2-Nitropropane induction. Likewise, MnSOD and catalase activity increased when groups P1, P2 and P3 were given *M. oleifera* fruits extract. These data indicate an increase in the activity of MnSOD ^36^ and catalase enzymes assisted by the presence of antioxidant candidates and expenses from the *M. oleifera* fruit extract. ^6^^,^^32^^,^^33^

Induction of 2-Nitropropane can cause liver cell damage and acute liver injury, which was characterized by increased levels of SGOT and SGPT. ^18^^,^^27^ When given *M. oleifera* fruits extract, there was a decrease in liver SGOT and SGPT levels in groups P1 and P2 compared to groups O1 and O2. These findings indicate that *M. oleifera* extract can be a potential antioxidant candidate in liver damage due to 2-Nitropropane induction. ^18^

This study was in line with Nafi, ^37^ Bilqis, ^19^ Anis, ^18^ Fiqih, ^21^ Rahmi ^38^ and Susantiningsih's ^38^ research that *M. oleifera* fruit extract has high antioxidant levels and deserves to be called a potential antioxidant candidate in 2-Nitropropane-induced obese male mice through the parameters. The findings of increased levels of MDA, SGOT and SGPT in liver tissue indicate that 2-Nitropropane induction causes acute liver injury. This research answers the objectives, to observe the contains of antioxidants *M. oleifera* fruits extract against liver injury by 2-Nitropropane induction in an obese male mice model through pre-clinical study.

## Conclusion

*M. oleifera* fruit extract was statistically proven to be used as a candidate for potential antioxidant against liver injury of induction 2-Nitropropane in obese male mice model by looking at the levels of MDA, GSH. 8-OHdG, Catalase activity, MnSOD activity and SGOT and SGPT levels. Antioxidant substances in *M. oleifera* fruit extract can significantly reduce oxidative stress in acute liver injury caused by 2-Nitropropane induction. The limitation of this research is the induction of 2-Nitropropane which requires special skills. Further clinical research on healthy human subjects is needed.

## Data Availability

Figshare: Moringa oleifera Fruit Extract As a Potential Antioxidant Against Liver Injury By 2-Nitropropane Induction In Obese Male Mice Model: Pre-Clinical Study. https://doi.org/10.6084/m9.figshare.19655757.v2. ^39^ This project contains the following underlying data:
• Data Moringa oleifera fruit.xlsx. ( *Moringa oleifera* fruit as a candidate for potential antioxidant against liver injury in the early events of chemically induced carcinogenesis, such as 2-Nitropropane in male obese mice models.) • Raws Data DokMar.xlsx (The data of MDA and GSH levels, 8-OHdG, MnSOD levels, SGPT and SGOT level). • Raw Data yang diminta Reviewer oleh MST TS.xlsx (Raw data for *Moringa oleifera* fruit as a candidate for potential antioxidant against liver injury in the early events of chemically induced carcinogenesis, such as 2-Nitropropane in male obese mice models.) Figshare: ARRIVE checklist for ‘ *Moringa oleifera* fruit extract as a potential antioxidant against liver injury by 2-Nitropropane induction in obese male mice model: pre-clinical study’ https://doi.org/10.6084/m9.figshare.19655757.v2. ^39^ Data are available under the terms of the Creative Commons Attribution 4.0 International license (CC-BY 4.0).
