# Supplementary material for: Moringa oleifera fruit extract as a potential antioxidant against liver injury by 2-Nitropropane induction in obese male mice model: pre-clinical study
Source: F1000Res. 2024 Feb 12;12:300. Originally published 2023 Mar 20. [Version 2] doi: 10.12688/f1000research.121695.2 (PMC11401981; doi:10.12688/f1000research.121695.2)
Supplement: Peer review history

# Reviewer report: Reviewer response for version 2

**DOI:** 10.5256/f1000research.162380.r246063

**Published:** 2024-09-14

**Authors:** Taghred Saber

**Affiliations:** 1 Zagazig University, Zagazig, Egypt

The author has done all the revisions in the manuscript.

Is the work clearly and accurately presented and does it cite the current literature?

Yes

If applicable, is the statistical analysis and its interpretation appropriate?

Partly

Are all the source data underlying the results available to ensure full reproducibility?

Yes

Is the study design appropriate and is the work technically sound?

Partly

Are the conclusions drawn adequately supported by the results?

Yes

Are sufficient details of methods and analysis provided to allow replication by others?

Partly

Reviewer Expertise:

Neurotoxicity, genotoxicity, Hepatotoxicity, Nephrotoxicity, Cardiotoxicity, Testicular toxicity

I confirm that I have read this submission and believe that I have an appropriate level of expertise to confirm that it is of an acceptable scientific standard.

# Reviewer report: Reviewer response for version 1

**DOI:** 10.5256/f1000research.133590.r209938

**Published:** 2023-10-18

**Authors:** Taghred Saber

**Affiliations:** 1 Zagazig University, Zagazig, Egypt

I found that this manuscript is totally relevant for the field and brings interesting results that deserve consideration. However, certain points need to be revised before the manuscript could be considered for indexing.

- The laboratory conditions of the animal house should be provided.
- The solvent *of moringa oleifera* extract should be provided.
- The level of statistical significance ( *P *value) should be mentioned under the title of Statistical Analysis.
- Statistical comparison should be provided in the results and foot note of the figures.
- The authors should mention the result values as mean ± standard error.
- Foot note of all figures should include the value of *P*.
- Figure legends are short and incomprehensible. It is necessary to expand and rewritten the legend for these figures.

Is the work clearly and accurately presented and does it cite the current literature?

Yes

If applicable, is the statistical analysis and its interpretation appropriate?

Partly

Are all the source data underlying the results available to ensure full reproducibility?

Yes

Is the study design appropriate and is the work technically sound?

Partly

Are the conclusions drawn adequately supported by the results?

Yes

Are sufficient details of methods and analysis provided to allow replication by others?

Partly

Reviewer Expertise:

Neurotoxicity, genotoxicity, Hepatotoxicity, Nephrotoxicity, Cardiotoxicity, Testicular toxicity

I confirm that I have read this submission and believe that I have an appropriate level of expertise to confirm that it is of an acceptable scientific standard, however I have significant reservations, as outlined above.

# Reviewer report: Reviewer response for version 1

**DOI:** 10.5256/f1000research.133590.r204636

**Published:** 2023-10-02

**Authors:** Wahyunia Likhayati Septiana

**Affiliations:** 1 Histology, Faculty of Medicine, Gunadarma University, Depok, West Java, Indonesia; 2 Program Doktor Ilmu Biomedik, Faculty of Medicine, Universitas Indonesia, Depok, West Java, Indonesia

1. The researchers mentioned using obese mice with a body weight of 40-60 grams. What data indicates that these mice were obese? Certain indicators are frequently used to assess the level of obesity and the impact of anti-obesity drugs. These variables include blood pressure, body mass index, insulin tolerance test, total body fat, adiposity index, Lee index, fat pad mass, and magnetic resonance imaging and dual-energy X-ray absorptiometry.
2. This study employed 2-Nitropropane to induce oxidative stress and liver injury in male obese mouse models. The study involved two groups, O1 and O2, which were given 2-Nitropropane once and twice. It is advisable to include the dosing schedule because it is not clear how much time passed between the two doses and how long after 2-Nitropropane and M. oleifera extract were administered.
3. 2-Nitropropane can induce liver injury. Is there data indicating that this induction was successful? Can the authors provide a histopathological description of the liver?
4. The results of this study, both from the graphs and the result descriptions, do not show which data is significantly different between groups. No p-values are mentioned. How can the authors claim that there are significant results?
5. What is the basis for administering a dose of 500mg/kgBW? Have there been any previous studies on this? The method section does not cite any previous research.
6. The GSH level decreased more in group P2 compared to O2. However, in group P1 with O1, this decrease was not as pronounced. Why did this happen? The same pattern also occurred in the levels of MDA, SGOT, SGPT, and 8oHDG. Please explain this in the discussion. This raises the question of whether M. oleifera might be more effective when given in cases of more severe liver injury.

Is the work clearly and accurately presented and does it cite the current literature?

Yes

If applicable, is the statistical analysis and its interpretation appropriate?

Partly

Are all the source data underlying the results available to ensure full reproducibility?

Partly

Is the study design appropriate and is the work technically sound?

Partly

Are the conclusions drawn adequately supported by the results?

Partly

Are sufficient details of methods and analysis provided to allow replication by others?

Partly

Reviewer Expertise:

Stem cells, Liver organoid, scaffold.

I confirm that I have read this submission and believe that I have an appropriate level of expertise to confirm that it is of an acceptable scientific standard, however I have significant reservations, as outlined above.

## References

[1] : Murine Models of Obesity. *Obesities* .2022;2(2) : 10.3390/obesities2020012 127-147 10.3390/obesities2020012

[2] : Chitosan nanoparticles as a promising candidate for liver injury induced by 2-nitropropane: Implications of P53, iNOS, VEGF, PCNA, and CD68 pathways. *Sci Prog* .2021;104(2) : 10.1177/00368504211011839 368504211011839 10.1177/00368504211011839 33940981 PMC10455010

# Reviewer report: Reviewer response for version 1

**DOI:** 10.5256/f1000research.133590.r204632

**Published:** 2023-09-26

**Authors:** Terisha Ghazi

**Affiliations:** 1 Discipline of Medical Biochemistry, School of Laboratory Medicine and Medical Sciences, University of KwaZulu-Natal, Durban, South Africa

In this study, Thadeus et al. investigated the antioxidant potential of Moringa oleifera fruit extract against 2-nitropropane-induced liver injury in obese male mice. The findings are interesting; however, there are some flaws that should be addressed:

1. In the methods section, the value of p that was considered statistically significant needs to be mentioned.
2. The authors mention throughout the manuscript that their findings were statistically significant but, in the results, there is no indication of the level of significance. The p values need to be included for each result obtained and the level of significance needs to be denoted by the use of "*, **, or ***" on the graphs.
3. Once the appropriate statistical significance is shown, the correct conclusions can be drawn. Statistical significance is required to verify the current conclusions made by the authors.
4. There are also many spelling and grammatical errors. The authors should have the manuscript proofread by an English language expert.
5. Mice and rats are not the same. Yet, it is used interchangeably in the manuscript. This needs to be corrected. 
6. In the discussion, the authors merely repeat their results. The results need to be discussed in the context of other previously published literature. Are the findings in this study similar or different from other published studies?

Is the work clearly and accurately presented and does it cite the current literature?

Yes

If applicable, is the statistical analysis and its interpretation appropriate?

No

Are all the source data underlying the results available to ensure full reproducibility?

Yes

Is the study design appropriate and is the work technically sound?

Yes

Are the conclusions drawn adequately supported by the results?

Partly

Are sufficient details of methods and analysis provided to allow replication by others?

Partly

Reviewer Expertise:

Biochemistry and molecular biology, including oxidative stress, DNA damage, and epigenetics.

I confirm that I have read this submission and believe that I have an appropriate level of expertise to confirm that it is of an acceptable scientific standard, however I have significant reservations, as outlined above.

# Response

**Published:** 2023-09-27

**Authors:** Maria Selvester Thadeus

**Affiliations:** UPN Veteran Jakarta, Indonesia

We greatly appreciate your constructive criticism.We hope that every bit of your suggestions will contribute to helping us better our future articles. Many thanks so much.

# Response

**Published:** 2023-10-06

**Authors:** Maria Selvester Thadeus

**Affiliations:** UPN Veteran Jakarta, Indonesia

Dear Reviewer

 We want to take this opportunity to let you know our sincere appreciation for your feedback. Suggestions and criticism are very constructive for our upcoming research and articles.

 Thank you very much.

 Best Regards

 Maria Selvester Thadeus
